# Opto-Mechanical and Electronic Design of a Tunnel-Trap Si Radiometer

**DOI:** 10.6028/jres.105.064

**Published:** 2000-12-01

**Authors:** George P. Eppeldauer, Donald C. Lynch

**Affiliations:** National Institute of Standards and Technology, Gaithersburg, MD 20899-8441 USA; Reyer Corporation, New Market, MD 21774

**Keywords:** detector, irradiance, photocurrent, photodiode, radiant power, reference detector, responsivity, spectral response, transfer standard

## Abstract

A transmission-type light-trap silicon radiometer has been developed to hold the NIST spectral power and irradiance responsivity scales between 406 nm and 920 nm. The device is built from replaceable input apertures and tightly packed different-size silicon photodiodes. The photodiodes are positioned in a triangular shape tunnel such that beam clipping is entirely eliminated within an 8 field-of-view (FOV). A light trap is attached to the output of the radiometer to collect the transmitted radiation and to minimize the effect of ambient light. The photodiodes, selected for equal shunt resistance, are connected in parallel. The capacitance and the resultant shunt resistance of the device were measured and frequency compensations were applied in the feedback network of the photocurrent-to-voltage converter to optimize signal-, voltage-, and loop-gain characteristics. The trap radiometer can measure either dc or ac optical radiation with high sensitivity. The noise-equivalent-power of the optimized device is 47 fW in dc mode and 5.2 fW at 10 Hz chopping. The relative deviation from the cosine responsivity in irradiance mode was measured to be equal to or less than 0.02 % within 5° FOV and 0.05 % at 8° FOV. The trap-radiometer can transfer irradiance responsivities with uncertainties comparable to those of primary standard radiometers. Illuminance and irradiance meters, holding the SI units (candela, color- and radiance-temperature), will be calibrated directly against the transfer standard trap-radiometer to obtain improved accuracy in the base-units.

## 1. Introduction

### 1.1 Light-Trap Standards

Light-trap detectors have been used as radiometric standards since 1983 [1]. At that time UDT UV100^1^ n-on-p inversion layer silicon photodiodes were used in either four element (Model QED-100) or three element (Model QED-200) reflectance-type light-trap configurations [2, 3]. These primary standard devices measured the total power of the incident, well collimated, radiation. Their power response relative standard uncertainty was 0.03 % between 440 nm and 460 nm where bias voltage was not applied to the photodiodes [4]. (Note that throughout this paper, all uncertainties are either relative standard uncertainties or standard uncertainties, i.e., one standard deviation estimates, and hence the coverage factor used is *k* = 1 [5].) These nonlinear devices had a limited dynamic range of operation [6].

Later, Hamamatsu S-1337 p-on-n silicon photodiodes were used in the Model QED-150 trap-detectors in an arrangement similar to the QED-200. These detectors were called “quantum-flat” because they have external quantum efficiencies (EQE) that are constant to within 0.1 % from 550 nm to 860 nm. The spectral responsivity of quantum detectors (in A/W) is proportional to their EQE and wavelength. The proportionality factor is *e/ħc*, where *e* is the elementary electron charge, *ħ* is the Planck constant, and *c* is the speed of light in vacuum. Using the silicon photodiode self-calibration technique [7] for a single element S-1337 photodiode, the quantum flatness of these trap-detectors could be extended to 400 nm [8]. The S1337 trap-detectors with the constant relative spectral responsivity were calibrated against either a QED-200 between 440 nm and 460 nm or an electrical substitution cryogenic radiometer. The responsivity of S1337 trap-detectors could be extrapolated from the 440 nm to 460 nm range to longer wavelengths with very little loss of accuracy because the shape of the internal quantum efficiency (IQE) does not depend on typical diode-to-diode variations in the doping profile. IQE is the ratio of the number of collected electrons to the number of photons absorbed by the detector after the front surface reflection loss. EQE = (1–*ρ*)IQE where *ρ* is the reflectance. Since the spectral shape of the IQE of S1337 type photodiodes can be modeled with very small uncertainty [9, 10], the spectral responsivity of S1337 reflectance-type trap-detectors could be interpolated between 406 nm and 920 nm with a relative standard uncertainty of 0.03 % if two or more absolute tie points were measured.

### 1.2 Reflectance-Type Trap Detectors

The reflectance-type (three-element) trap detectors have polarization-dependent fractional response variations of about 1 × 10^−4^ [11]. The reflectance loss of the S-1337 reflectance-type trap detectors increases from 0.21 % at 920 nm to 1 % at 406 nm [10]. We measured the responsivity ratios of a QED-150 to a S1337 reflectance-trap versus wavelength. The ratio changed from 1.0005 at 920 nm to 1.0085 at 406 nm, indicating that the reflectances in the blue for the same S1337 photodiode model can be different by several tenths of a percent. The reflectance depends on the oxide thickness of the selected S1337 photodiode. A spatial response non-uniformity of 0.03 % was measured on the same S1337 reflectance-trap where the photodiodes were individually aligned to optimize the device field-of-view (FOV) [12]. Later, 0.2 % spatial response non-uniformities were measured on three different QED-150 devices using the same characterization facility [13]. The measured active areas of the QED-150 trap detectors were neither symmetrical nor similar. The higher response non-uniformities and the different shapes of the measured areas indicated that either the FOV was smaller and non-symmetrical or the reproducibility of the photodiode device to device positioning was poor.

Typically, trap detectors have a small FOV. On a QED-150 we measured a 4° FOV when it was equipped with a 3.5 mm diameter aperture. The response deviation from the cosine function was 0.2 % in this angular range. The alignment of reflectance-type trap detectors can be difficult when measuring non-parallel beams. The beam convergence-angle should always be less than the FOV of the trap detector to avoid beam clipping on the photodiodes. It is easy to obtain a false maximum in the output signal owing to reflections from shiny surfaces other than the active areas of the photodiodes.

At present, trap detectors are not commercially available. Also, the characteristics described above of reflectance-type trap detectors show that they should be significantly improved to transfer the 0.01 % to 0.03 % relative standard uncertainties of primary standard radiometers with a minimum loss of accuracy. Our measurement-transfer policy is to shorten the scale derivation chain by making direct responsivity calibrations against transfer standard radiometers that have uncertainties similar to those of the primary standards.

### 1.3 Transmission-Type Trap Detectors

Tunnel-trap detectors were reported several years ago that were constructed using four [14] and six photodiodes [15]. Because they were free of back reflectance, separately calibrated filters or other optical components could be attached to their inputs without introducing additional uncertainties. The six-element version had a polarization independent responsivity [11, 14], lower reflectance loss, and improved spatial response uniformity. Because of these advantages, the six-element transmission-type trap-radiometer is the best choice to transfer responsivity with the lowest possible uncertainty. In order to make the transfer for an extended wavelength range, we wanted to utilize the physical model for IQE of S1337 photodiodes. In addition to traditional radiant power mode measurements, irradiance mode measurements are needed within a larger FOV for an extended range of applications. The sensitivity had to be increased because the collected flux in irradiance measurements is much smaller than in power mode where the total power in the beam is measured. The responsivity uncertainty in irradiance mode also had to be similar to the uncertainties of the primary standards.

The angular response of the transmission-type trap detector we developed earlier was measured. Here six equal size photodiodes were used and the aperture diameter was 3.5 mm. In this experimental device, the positioning of the photodiodes was not designed and implemented carefully enough. The angular response of this device (No. 01 trap) will be shown below. The FOV was about 4° and the deviation from the cosine function was about 0.1 % in this angular range. The asymmetry was caused by beam clipping on the sixth photodiode.

The design goal for the new tunnel-trap radiometer was to achieve a deviation of less than 0.02 % from the cosine function in an angular range of 5° minimum. A signal dynamic range of eleven decades is needed to perform irradiance measurements over seven decades with a signal to noise ratio of 10^4^. In order to achieve this requirement, a noise equivalent photocurrent of 0.1 pA had to be achieved.

### 1.4 Photocurrent Meters

A high accuracy current meter is necessary to measure the photocurrent. The lowest uncertainty of commercially available current meters is 0.03 %. The lowest signal level where this uncertainty can be achieved is 0.1 μA. Higher sensitivity current preamplifiers have gain temperature coefficients of 0.022 %/°C at a gain selection of 10^9^ V/A. These photocurrent meters require ambient temperature control to within 0.5 °C to perform current-to-voltage conversion close to the 0.01 % relative standard uncertainty of trap radiometer based measurement transfer. Also, these preamplifiers require a source resistance (photodiode shunt resistance) greater than the feedback resistance used to achieve the specified current-to-voltage conversion uncertainty. For instance, with a sensitivity selection of 1 nA/V, a source resistance larger than 1 GΩ is needed. A 3.37 % uncertainty from systematic effects (Type B) [5] was obtained in the current-to-voltage conversion at this preamplifier gain selection when the photocurrent of the 31 MΩ shunt resistance trap detector was measured.

A procedure had to be introduced to select and group different size Hamamatsu S1337 photodiodes according to shunt resistance and junction capacitance. The photocurrent meter for a photodiode group had to be optimized to achieve an adequate noise floor and a 0.01 % relative standard uncertainty in the current-to-voltage conversion.

Discussed in Sec. 2 of this paper is a precise optical-mechanical design that made it possible to fabricate devices of equal performance. Section 3 describes a detailed electronic design and circuit implementation that was necessary to achieve the electronic characteristics described above.

## 2. Opto-Mechanical Design

Medium and large size silicon photodiodes, equivalent to the S1337 model, were selected to utilize the physical model for spectral responsivity extrapolation and interpolation. Also, by choosing different size photodiodes, the aperture area and the FOV could be maximized.

### 2.1 Tunnel-Trap Arrangements

Different arrangements of six photodiodes were investigated to determine the most suitable geometry for construction in terms of ease of fabrication, accuracy of mounting surfaces, and optimization of the aperture size and FOV. The FOV is dependent on two parameters: the total path length from the aperture to the last detector and the size of the aperture placed in front of the first detector. To obtain a large FOV, the total path length must be kept as short as possible.

**Fig. 1:**
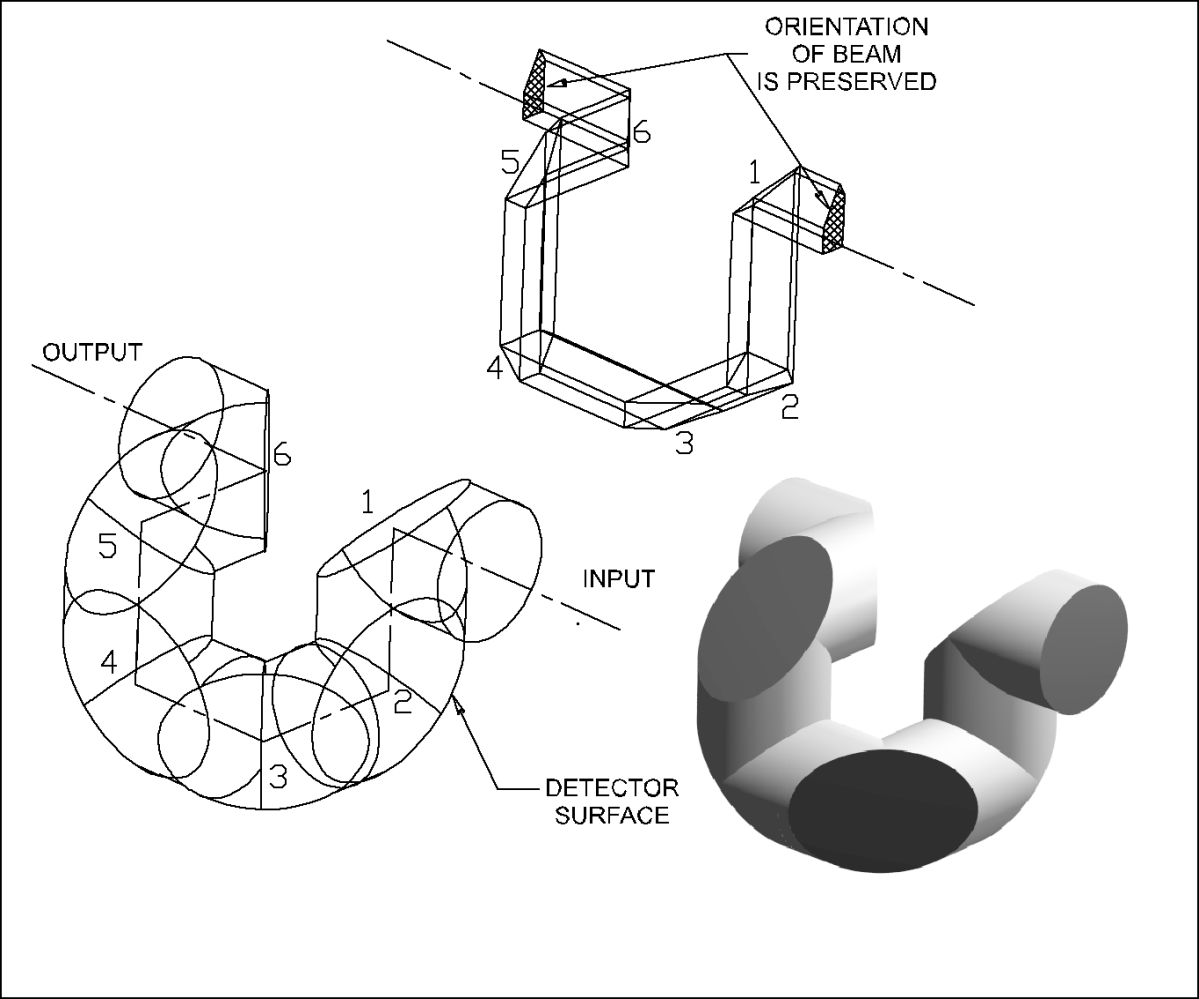
Generic transmission trap detector arrangement. The input and output beams are collinear.

The analysis began with the polarization-independent arrangement shown in Fig. 1, where six detectors are arranged such that the input and output beams are collinear [14]. The lower left portion of the figure depicts a parallel cylindrical beam being reflected from the six detector surfaces. As can be seen from the upper portion of the figure, the input and output beams have the same orientation. The total path length is at a minimum when the detectors begin to touch each other. In the early stages of the development, it became apparent that using detectors of two different sizes could shorten the path length. Smaller detectors can be used in the first two positions because a diverging beam (focused onto the aperture) covers only small portions of these two detector surfaces.

The arrangement shown in Fig. 1 was rejected for two reasons. First, it is difficult to produce an accurate holder for the detectors. Second, the first and sixth detectors mechanically interfere with each other when using the two different size detectors unless the path length is increased, which would reduce the FOV.

**Fig. 2:**
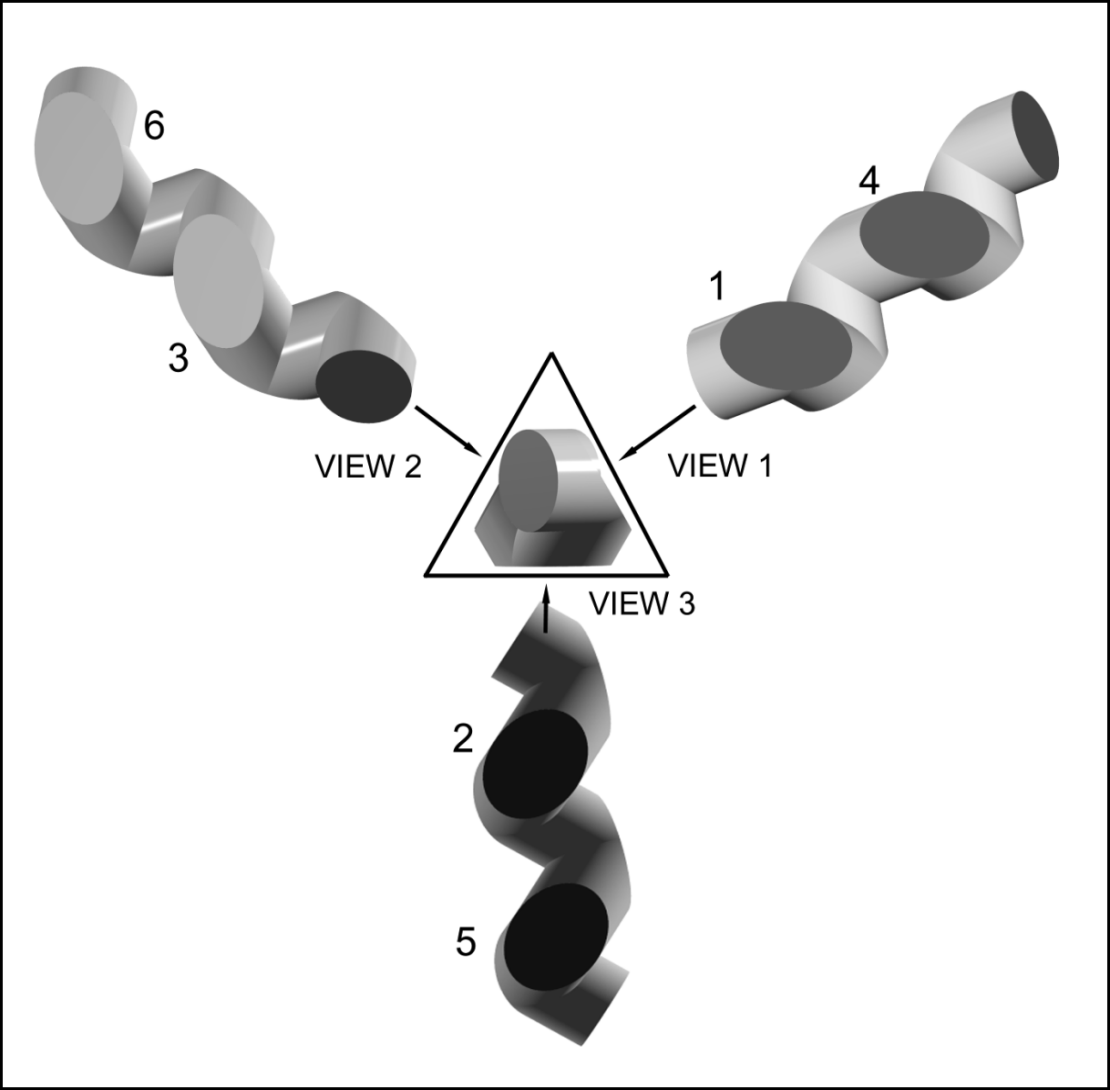
The triangular tunnel-trap detector where the last three detectors of the generic arrangement are rotated by 180°.

Many detector arrangements can be produced by starting with the generic arrangement shown in Fig. 1 and simply rotating one, two, or three adjacent detectors at a time about a portion of the optical axis that lies between two adjacent detectors. Of all the detector arrangements that were produced by rotating various groups of detectors, only two arrangements preserved the input and output beam orientation: the arrangement in Fig. 1 and that shown in Fig. 2. The arrangement in Fig. 2 was derived from that shown in Fig. 1 by rotating detectors numbered four through six about the axis between the third and fourth detector centers by 180°. Here, for simplicity, all six detectors are of the same size.

### 2.2 Triangular Tunnel-Trap

As can be seen from Fig. 2, the central view has a triangular-like shape, where views 1 through 3 show that each side of the triangle contains two detector surfaces. Each pair of detectors forms the side of an equi lateral triangle and two pairs form planes with a dihedral angle of 60°. This tunnel-trap arrangement, which will be called the triangular tunnel-trap, was selected because it allowed a simple method of construction and minimized the total path length.

**Fig. 3:**
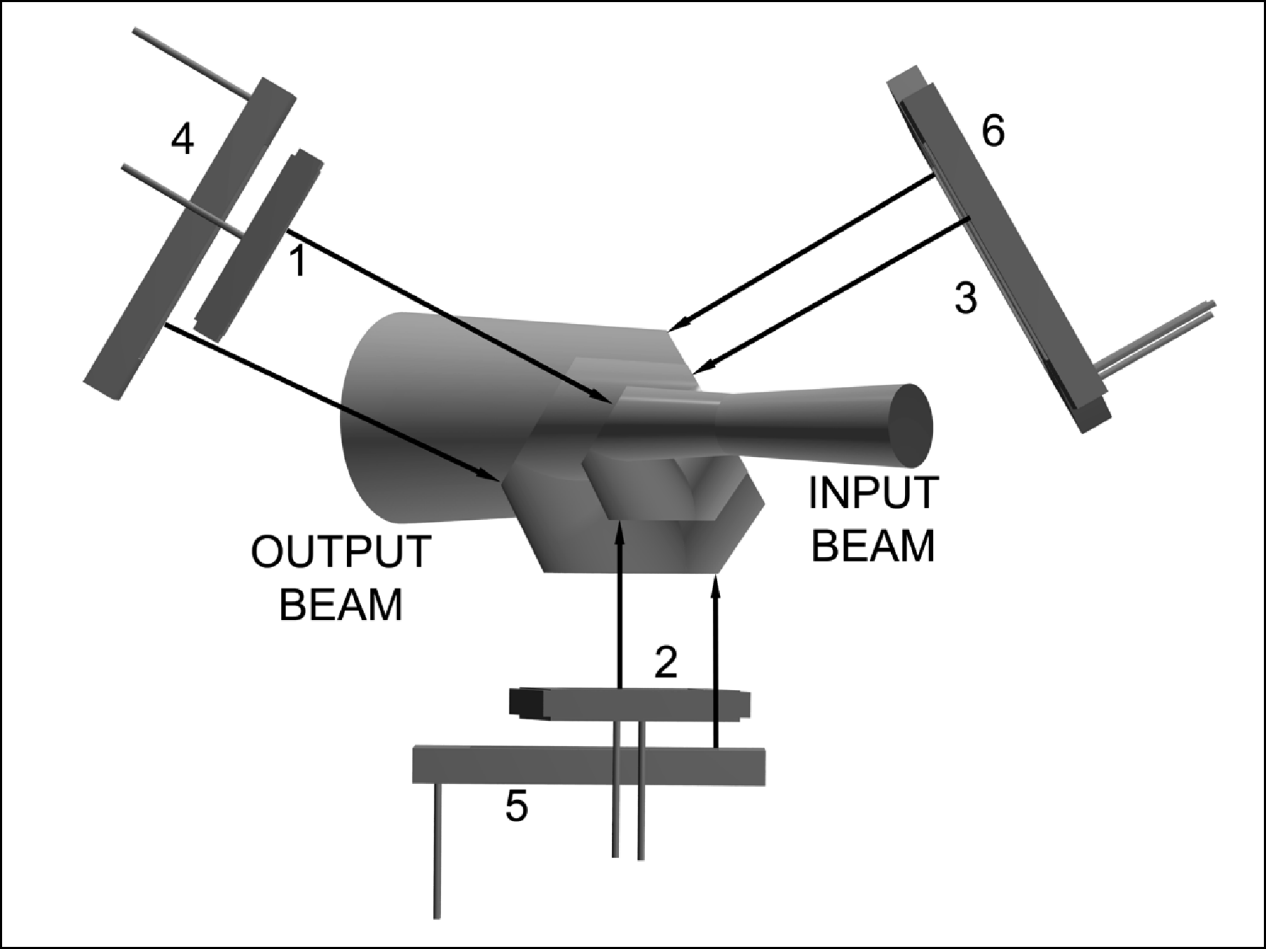
Photodiode arrangement and beam geometry in the triangular tunnel-trap detector. The input beam hits photodiode 1 and then it propagates to photodiodes 2 to 6.

The triangular tunnel-trap detector is shown in Fig. 3 with a 6° diverging beam reflecting off the planes of the detector surfaces of two different sizes. The detectors are shown offset from the beam path to clearly show how the planes of the detector pairs of different sizes are parallel, but do not lie in the same plane, whereas the detectors of the same size (shown in Fig. 2) lie in the same plane.

**Fig. 4:**
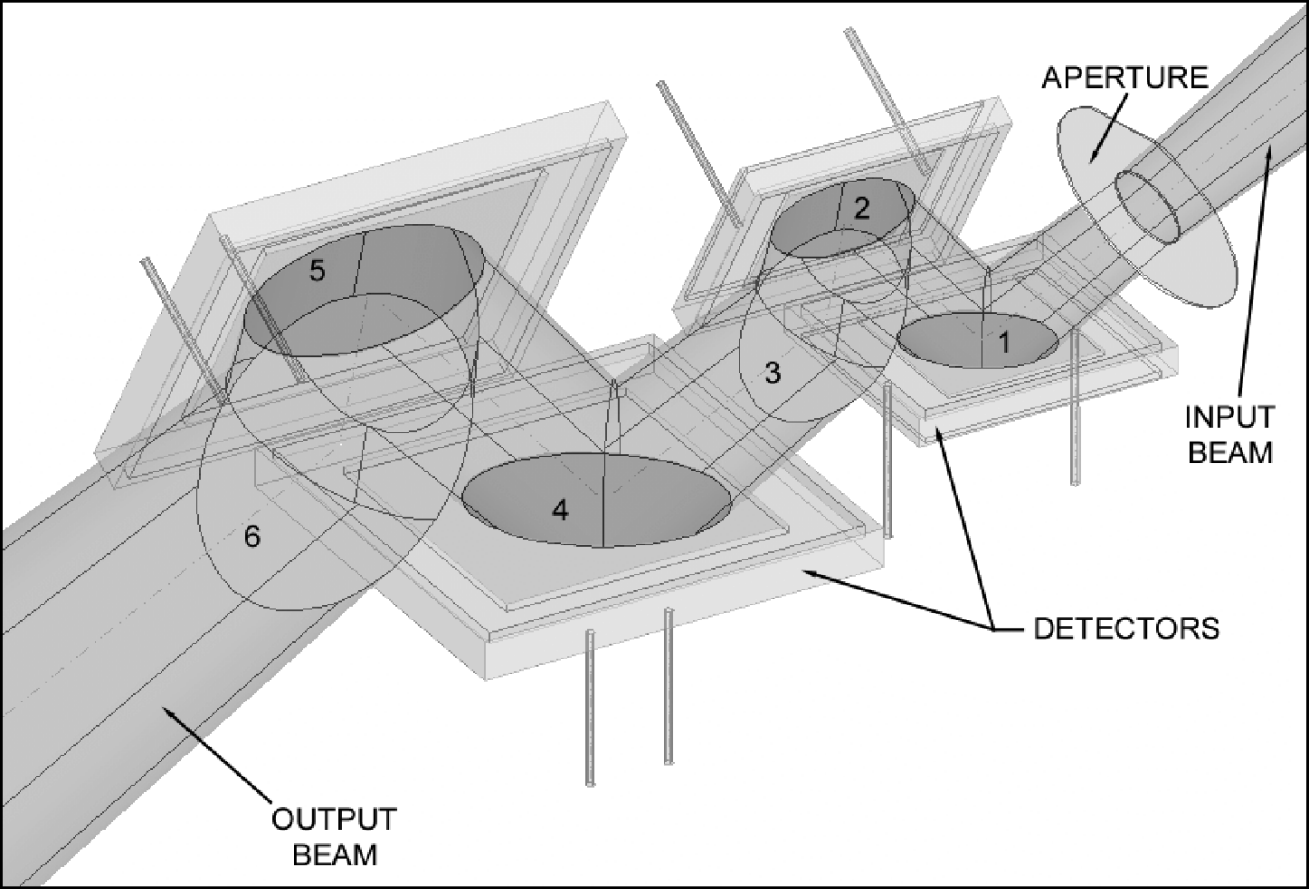
Photodiode arrangement and beam propagation in the triangular tunnel-trap detector.

The final photodiode arrangement is shown in Fig. 4. In this arrangement the detectors numbered three and six were left out so the placement of the detectors can be seen. The positions of both the photodiodes and the aperture were obtained from ray-tracing an *f*/4 incident beam focused on and normal to the reference plane of the radiometer. The reference plane, needed for irradiance measurements, is the front surface of the aperture. The diverging beam leaving the opening of the overfilled aperture was collected and measured by all of the photodiodes to maintain the cosine function of irradiance responsivity measurements. The input and output beams lie in the same plane which is parallel to the base plane of the radiometer housing. The triangular holder is mounted in this plane which is also parallel to the front surfaces of photodiodes two and five. The output beam is shifted parallel relative to the input beam by 41.3 mm. This shift does not cause any measurable polarization dependence in the signal responsivity of the trap radiometer. The figure also shows that the first and second detector surfaces are more efficiently utilized and the position of detector four as well as of detector three is not as critical.

The last detector was rotated in the plane of the block to maximize the detector area with respect to the elliptical shape of the beam.

### 2.3 Tunnel-Trap Construction

**Fig. 5:**
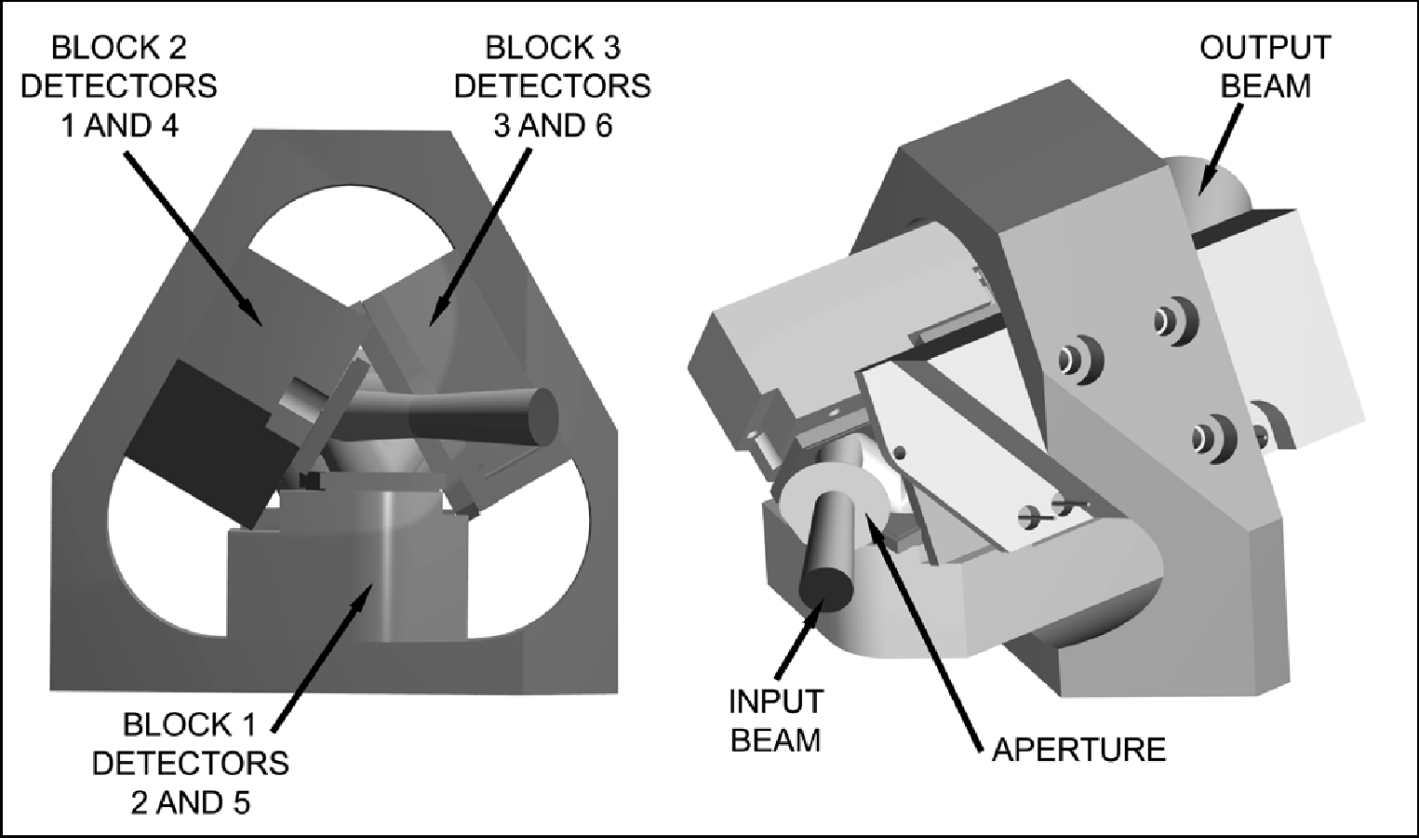
Construction of the triangular tunnel-trap detector holder.

The construction, shown in Fig. 5, is made up of three blocks arranged in a triangular fashion. Six silicon photodiodes of two different sizes were used as detectors in the tunnel-trap radiometer. Two 10 mm by 10 mm photodiodes at the front were followed by four 18 mm by 18 mm devices. Windowless photodiodes were used to eliminate problems caused by additional reflections and laser interference. There are two blocks that hold detectors of two different sizes and therefore have a step to allow the smaller detector to be closer to the center of the triangle. The third block holds the larger detectors at the same distance from the center of the triangle. The three blocks are held in a triangular shaped block that has been ground both on the inside and outside to achieve flatness and thickness accuracy within a few hundredths of a millimeter. This holder simplified the alignment of the photodiodes before installation inside of the radiometer housing.

**Fig. 6:**
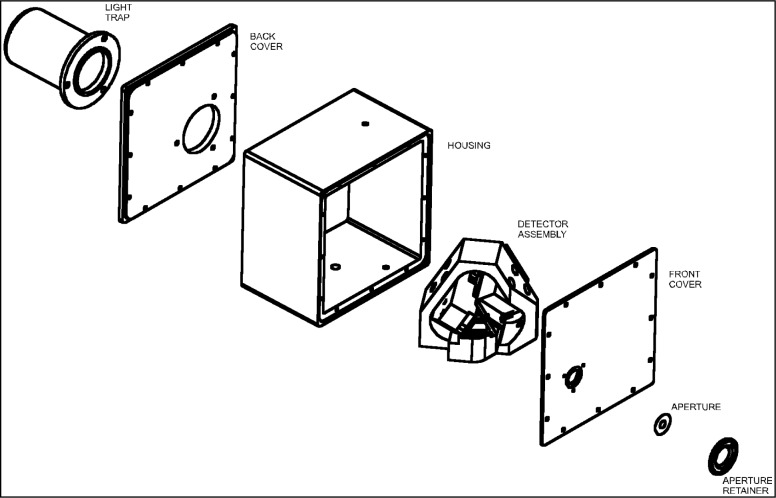
Exploded view of the triangular tunnel-trap radiometer.

The triangular shaped detector assembly block is housed in a square housing as shown in an exploded view of the triangular tunnel-trap radiometer (Fig. 6). Two precision apertures, with internal diameters of 5 mm and 3.5 mm and made of black-nickel coated copper disks 0.1 mm thick, can be applied at the input alternatively. They are mounted in a recess 0.1 mm deep in the front cover to extend the aperture front plane to the overall front surface of the trap-radiometer. This arrangement facilitates the distance measurements in the irradiance mode. The aperture mounting positions are invariant. The FOV is 6° with the 5 mm aperture and 8° with the 3.5 mm aperture. The installed aperture is fixed by a cylindrical holder threaded outside for either a protecting cap or an input baffle tube. The aperture retainer is beveled inside at the front with an angle of 45° relative to the aperture plane. This input geometry minimizes the stray radiation at the front of the radiometer. The incident radiation, overfilling the aperture, is reflected back from the front surfaces such that the reflected beams cannot enter the aperture opening. A light trap can be attached to the radiometer output to reject the ambient light from the trap detector. This can keep the blocked signal reading and the signal fluctuations low. The light trap and the protecting cap are attached to the housing most of the time to keep the dust outside. When the light trap is removed, the transmitted radiation can be measured. Also, the transmitted light can expedite the radiometer alignment. The cavity of the light trap is covered with specularly reflective black paint.

### 2.4 Optical Performance Test

The tunnel-trap radiometer built according to the above design considerations was tested for optical performance. Angular response scans were made by rotating the radiometer around the center of its 3.5 mm diameter aperture. The output signal was measured while the aperture was overfilled with the uniform irradiation from a point source. The source was a stable and low noise Wi-41G tungsten lamp located at a distance of 3 m from the aperture.

Since the projected area of the rotating aperture changes with cosine versus rotation angle, the irradiance response, which is proportional to the projected area, also should follow the cosine function. Deviations from the cosine response can be caused by internal reflections, beam clipping, stray radiation, and angular response dependence of the under-filled photodiodes. When the source is different from a point source, the accuracy of irradiance measurements strongly depends on the deviation between the realized angular response and the cosine response within the angular range determined by the source size, the diameter of the detector aperture, and the separation between source and aperture. The radiometer FOV gives the final limitation for the angular range of an irradiance measurement. The FOV has a major impact for the maximum source size and/or the minimum source to aperture distance.

**Fig. 7:**
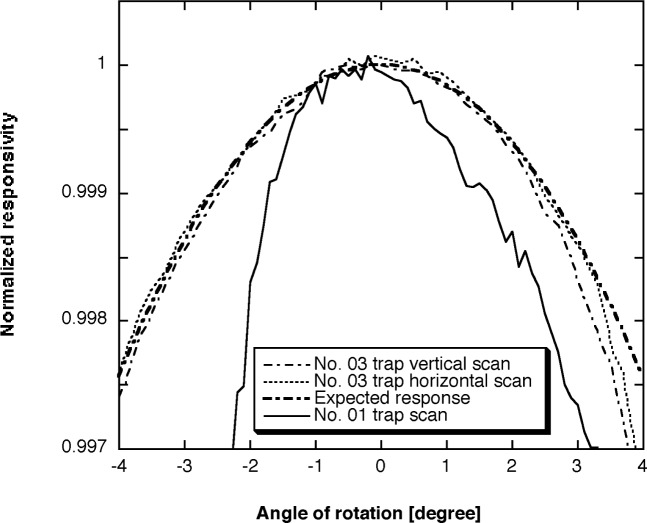
Irradiance mode angular responses of tunnel-trap radiometers using 3.5 mm diameter apertures. The experimental No. 01 trap was built with six equal size photodiodes. The beam was clipped on the sixth (last) photodiode. The horizontal and vertical angular response scans of the optimized tunnel-trap radiometer No. 03 are very close to the expected cosine response within an angular range of 5°.

The normalized angular responses of the experimental No. 01 trap discussed earlier and the optimized No. 03 tunnel trap radiometers are shown in Fig. 7 and compared with a perfect cosine distribution. During the horizontal scan, radiometer No. 03 was rotated in the horizontal plane around its aperture center. Thereafter, it was rotated 90° around its optical axis and the “vertical” scan was done in the same way as the horizontal one. The graph shows that the deviation of the realized angular response from the expected cosine function is less than 0.02 % within a 5° FOV and about 0.05 % at 8° FOV. The cosine response of the optimized device is five times better than that of the experimental device where six equal-size photodiodes were used. Also, the FOV increased significantly. These measurement results verified the optical and mechanical design expectations.

## 3. Electronic Characteristics

To optimize the overall performance of the tunnel-trap radiometer, the photocurrent-to-voltage converter had to be optimized for the three fundamental gains: current-, voltage-, and loop gain [16].

The impedance of the feedback components determines the photocurrent gain and the high frequency signal roll-off [16]. A high resistance feedback resistor gives high signal gain and can produce a low signal roll-off frequency even with a small feedback capacitance.

The loop gain of the photocurrent meter is the product of the open loop gain of the operational amplifier and the attenuation of the feedback network. The feedback network is a signal attenuator from the converter output to its input, implemented by the feedback impedance and the shunt impedance of the photodiode. The loop gain is frequency dependent. It is very important to keep the loop gain high enough at the signal frequency because the accuracy of the current-to-voltage conversion depends on its magnitude [16].

The closed loop voltage gain is equal to the reciprocal of the feedback attenuation of the converter if the loop gain is high enough [16]. The voltage gain depends on the signal frequency. The voltage gain determines the amplification for the input noise and the temperature-dependent offset-voltage of the operational amplifier. The voltage gain can be kept low if photodiodes with high shunt resistance are used. When ac signals are measured, the voltage gain has to be low at the selected signal frequency.

In order to optimize the fundamental gains, the S1337 photodiodes had to be selected for shunt resistance and the resultant junction capacitance of the parallel connected photodiodes had to be measured. Selection of the different size photodiodes for equal shunt resistance [17, 18] was necessary to maximize the resultant shunt resistance of the six diodes connected in parallel.

The medium and large photodiodes were selected from several groups purchased at different times. The two photodiodes located directly behind the aperture were Model 1337-11 photodiodes with a 10 mm by 10 mm active area. The diode shunt resistances varied between 275 MΩ and 325 MΩ in the first group and between 130 MΩ and 267 MΩ in the second group. The last four diodes in the trap arrangement were Model 6337-01 photodiodes with an active area of 18 mm by 18 mm. The shunt resistances of these large area photodiodes varied between 355 MΩ and 500 MΩ in the first group and between 150 MΩ and 260 MΩ in the second group. Two 1337-11 and four 6337-01 photodiodes of similar shunt resistance were selected and connected in parallel in one radiometer. Eight such photodiode groups were assembled for the eight trap radiometers we built. Resultant shunt resistances between 30 MΩ and 55 MΩ were measured on the eight radiometers. A shunt resistance of 31 MΩ was measured on trap radiometer No. 03, which is characterized in detail in this paper.

In order to know the impedance of the parallel connected six photodiodes, the resultant capacitance of each photodiode group was measured. The capacitance of radiometer No. 03 was 7.5 nF in the dark.

### 3.1 Loop-Gain Characteristics

The loop gain at the signal frequency has to be high enough for all signal gain selections of the current-to-voltage converter to obtain a small enough uncertainty in the current-to-voltage conversion. For example, to achieve a 0.01 % uncertainty in a dc mode current-to-voltage conversion, a minimum loop gain of 10^4^ is required for signal frequencies lower than 0.3 Hz (see below). Because the loop gain is the product of the amplifier open-loop gain and the attenuation of the feedback network, it is also dependent on signal gain and thus on the feedback resistor. Care should be taken when high sensitivities are needed because increasing feedback resistance results in decreasing loop gain [16]. Frequency and feedback resistor dependent optimization of loop gains is one of the most important electronic design considerations in high accuracy photocurrent measurements and will be discussed in detail below.

The theory of loop-gain calculations in silicon radiometers has been described in a previous work [16]. The relatively small shunt resistance of the tunnel-trap detector can cause a large attenuation in the feedback network especially at high feedback resistor selections. The large attenuation can result in low loop gains in the current-to-voltage converter. The feedback network in this case was implemented by the complex impedance of the six photodiodes connected in parallel and that of the feedback resistance and capacitance connected in parallel. Also, the large 7.5 nF capacitance of the parallel-connected photodiodes increased the integrating-type time constant in the analog control loop. Because of these two reasons, oscillations occurred in the output voltage of the current-to-voltage converter even at the highest signal-gain settings. In order to eliminate loop oscillations and to achieve 0.01 % current-to-voltage conversion uncertainty and stability, the loop-gain characteristics of the trap radiometer had to be analyzed and optimized versus frequency and feedback-resistor for both dc and ac mode optical radiation measurements.

**Fig. 8:**
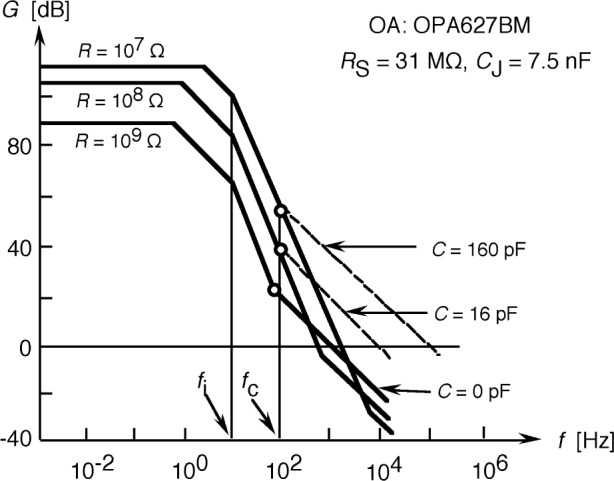
Calculated loop gain characteristics of trap-radiometer No. 03 for the highest three signal-gains. The open-loop gain roll-off frequency of the operational amplifier is *f*_i_. The dashed lines show the effect of the partial frequency compensations at frequency fc. The open circles show the signal 3 dB roll-off points matched to the loop gain curves.

The calculated loop gain characteristics of the tunnel-trap radiometer for the highest three signal gain selections are shown in Fig. 8. The feedback resistor *R* could not be higher than 1 GΩ to obtain a minimum dc loop gain of 10^4^ (80 dB). The solid lines show the loop gain curves when no external capacitors *C* are connected parallel to the feedback resistors. For 10^7^ Ω and 10^8^ Ω feedback resistor selections, two roll-off points can be seen on both curves above the 0 dB line causing a 180° phase shift lag at high frequencies. The roll-off points are caused by integrating type time constants. The larger time constant τ_1_ is equal to the parallel connected photodiode shunt resistance and feedback resistance times the sum of the detector and feedback capacitance [16]. The shorter time constant τ_i_ = 1/*f*_i_ is produced by the open-loop gain roll-off of the operational amplifier. The additional 180° phase shift, caused by the negative feedback itself, produces a total of 360° phase shift resulting in oscillations at high frequencies in the loop. At signal-gain selection 10^9^ V/A, the τ_2_ differentiating time constant produces the roll-on (roll-back) in the loop gain. This time constant, produced by the feedback resistor and the parallel stray capacitance, is large enough to decrease the 180° phase lag caused by the two integrating time constants. At this signal gain selection, oscillations will not happen if an operational amplifier with high enough open-loop gain and roll-off is selected. Our choice was the OPA627BM for which the open-loop gain is 10^6^ and the roll-off of the open-loop gain is at 10 Hz. In contrast, the roll-off is at 1 Hz for the popular OPA111.

**Fig. 9:**
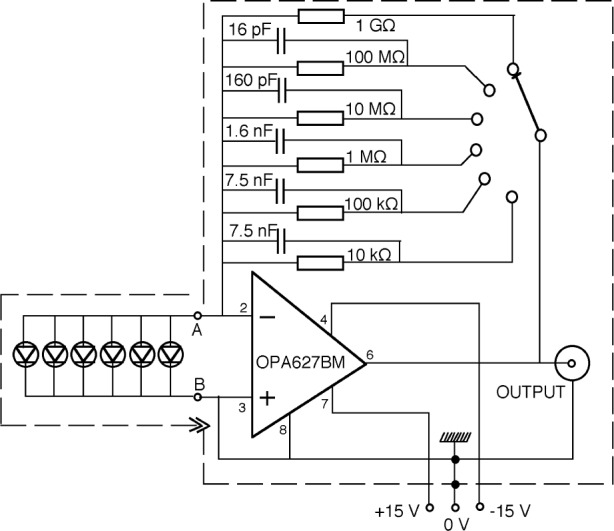
Circuit diagram of the tunnel-trap radiometer.

Frequency compensations can be applied in the feedback network of the operational amplifier to modify loop-gain characteristics [16]. Partial frequency compensations have been applied for the next three signal gains of the trap radiometer to eliminate oscillations. When an external capacitor of 160 pF was connected parallel to the 10^7^ Ω feedback resistor and a 16 pF to the 10^8^ Ω resistor, the signal roll-off points were tuned to *f*_c_ = 100 Hz (shown with open circles) and the phase lag was decreased enough to eliminate oscillations. Similarly, a compensating capacitor of 1.6 nF was connected parallel to the feedback resistor of 10^6^ Ω. Full frequency compensations were applied for feedback resistor settings of 10^5^ Ω and 10^4^ Ω. In a full compensation, the differentiating time constant of the loop was equalized to one of the loop integrating time constants. This integrating time constant was produced by the parallel connected photodiode shunt resistance and feedback resistance times the sum of the detector and feedback capacitances [16]. The values of the feedback components and the final signal roll-off frequencies of the trap radiometer are shown in Table 1. The circuit diagram of the trap radiometer is shown in Fig. 9. To minimize 60 Hz ripple and noise pick-up, the length of the wires, connected to the high-resistance inverting-input of the operational amplifier, was minimized and both the circuit board and the photodiodes were shielded. The temperature coefficient of the feedback resistors is equal to or less than 10^−4^ *R*/°C.

The optical radiation signal can be measured in either dc or ac (chopped) modes. In the dc mode, if the integration is done for the duration of 100 power line cycles, the electrical bandwidth is limited to 0.3 Hz. In this frequency range, the loop gain is 3 × 10^4^ or higher for any signal gain selections. However, the upper frequency limit is determined by the integration time of the digital voltmeter (DVM) attached to the output of the current-to-voltage converter. Frequency compensations are still needed to avoid oscillations in the analog control loop of the current-to-voltage converter.

In the ac mode, the chopping frequency was selected to be 10 Hz, a decade lower than the 3 dB roll-off points, to obtain operating points on the plateau of the frequency-dependent signal gain curves. To achieve high current-to-voltage conversion accuracy in the ac mode as well, the loop gain had to be high enough at 10 Hz for all signal gain selections. The lowest 10 Hz loop gain was equal to about 3 × 10^3^ at the highest signal gain (*R* = 10^9^ Ω). This loop-gain resulted in the largest current-to-voltage conversion uncertainty, namely, 0.03 %.

### 3.2 DC Mode Measurements

**Fig. 10:**
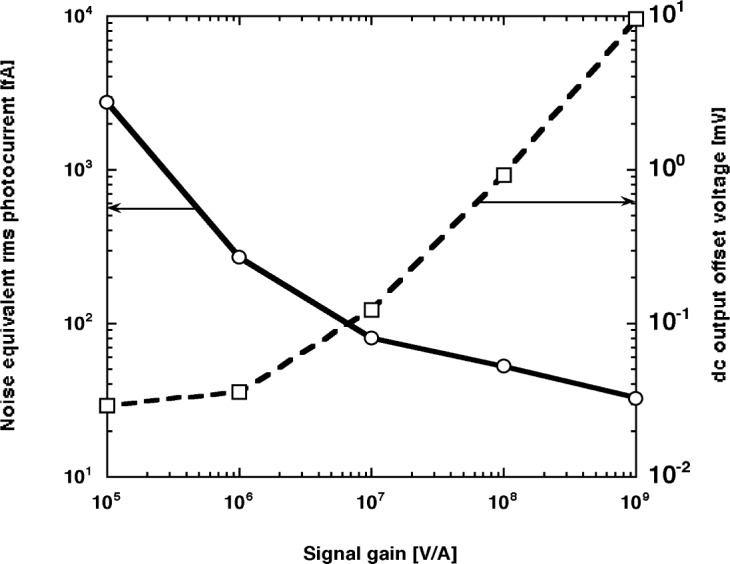
DC noise and output offset voltage measurements in the dark versus signal gain.

Figure 10 shows the dc noise measurements in the dark versus signal gain selections. The electrical bandwidth, determined mainly by the integrating type DVM, was 0.3 Hz. The noise equivalent rms photocurrent is shown with the solid line. The noise floor at 10^9^ V/A signal gain was 32.2 fA. This corresponds to a noise equivalent power of 47.4 fW at 845 nm where the responsivity of the trap detector is 0.6794 A/W. The graph also shows the dc output offset voltage of the current-to-voltage converter. The dc output offset voltage can be subtracted together with the dark reading if a shutter is applied at the light source being measured by the trap radiometer.

The noise sources in the dc mode photocurrent meters were discussed in an earlier work [17, 18]. The Johnson (thermal) noise, which is caused by thermal motions of charge carriers in resistive circuit elements, could be calculated for the trap radiometer. At the highest signal gain, the source resistance, which is the parallel connection of *R*_S_ = 31 MΩ and *R* = 1 GΩ, is *R*_SO_ = 3 × 10^7^ Ω. The 31 MΩ shunt resistance dominates the source resistance at the highest two signal gains. The rms noise voltage of the source resistance, at the output of the trap radiometer, is

$$V_{N}={\left(4kTR_{\text{SO}}\Delta f\right)}^{1/2}=0.39\;\mu V,$$ (1)

where *k* = 1.38 × 10^−23^ J/K is the Boltzmann constant, *T* = 300 K is the temperature of the radiometer during the test, and Δ*f* = 0.3 Hz is the measurement bandwidth determined by the DVM. The 0.39 μV is equivalent to an rms photocurrent of 0.39 fA. (The Johnson noise at *R* = 0.1 GΩ is 0.34 μV.)

The total noise of 32.2 fA measured in the dc mode indicates that the dominant noise originated from the 1/*f* noise of the operational amplifier. For 1/*f* noise, the noise power goes inversely as the frequency. The 1/*f* noise is very large in the measured frequency interval, that is, below 0.3 Hz. The signal frequency had to be increased to get closer to the elbow of the 1/*f* noise spectrum. This way the 1/*f* noise contribution could be decreased.

For the photocurrent measurements made in the dc mode, the effect of operational amplifier drift has to be taken into consideration as well. The drift in the output voltage of the current-to-voltage converter originates from two sources. The first is the amplified offset voltage drift of the operational amplifier. At a signal-gain selection of 10^9^ V/A, it will be 26 μV for a temperature change of 1 °C (0.8 μV/°C times the dc voltage gain of 33). The other drift component is the roughly 0.02 pA/°C offset current drift that produces an output voltage of 20 μV through the same feedback resistor for the same temperature change. The signal-produced voltage at the output of the current-to-voltage converter should be always 10^4^ times larger than the resultant output drift voltage to achieve 0.01 % measurement uncertainty. In a worst case situation, the output signal should not be lower than 0.46 V for a temperature change of 1 °C. This signal requirement applies for the output noise component of the converter as well. The 0.46 V output signal is also more than 10^4^ times larger than the measured 32.2 μV output noise (corresponding to the 32.2 fA noise discussed above). If the temperature of the operational amplifier is regulated to 0.1 °C, the drift components in the output voltage of the converter will be lower than 5 μV and the noise will limit the output signal at about 0.3 V.

### 3.3 AC Noise Measurements

**Fig. 11:**
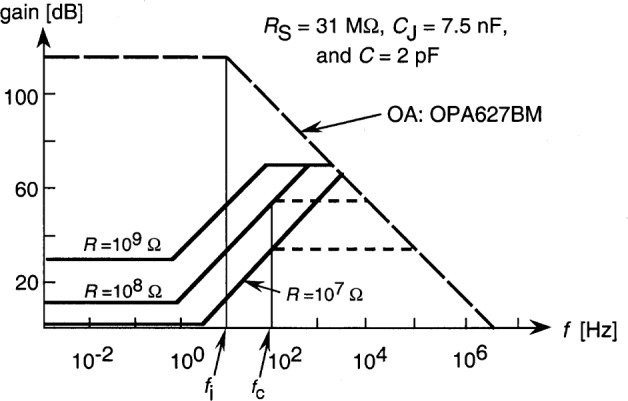
Calculated voltage gain characteristics of the trap radiometer. The open-loop gain roll-off frequency of the operational amplifier is *f*_i_. The short dashed lines show the effect of the partial frequency compensations at frequency *f*_c_.

According to the calculated voltage-gain curves in Fig. 11, trap radiometer No. 03 has a voltage-gain increase with increasing signal frequency for input 1/*f* voltage-noise between 1 Hz and 100 Hz. This is the frequency interval where ac measurements can be made. The voltage-gain curves are shown with solid lines for the highest three signal-gains. In each voltage-gain curve τ_1_ is a differentiating time constant producing a roll-on and τ_2_ is an integrating time constant producing a roll-off [16]. The long-dashed curve shows the frequency-dependent open-loop gain of the OPA627BM operational amplifier which limits the voltage gain at high frequencies. The frequency *f*_i_ shows the roll-off of the amplifier open-loop gain curve. The short-dashed lines show how the partial frequency compensations discussed above (at *f*_c_ = 100 Hz) can modify the shape of the voltage-gain curves.

**Fig. 12:**
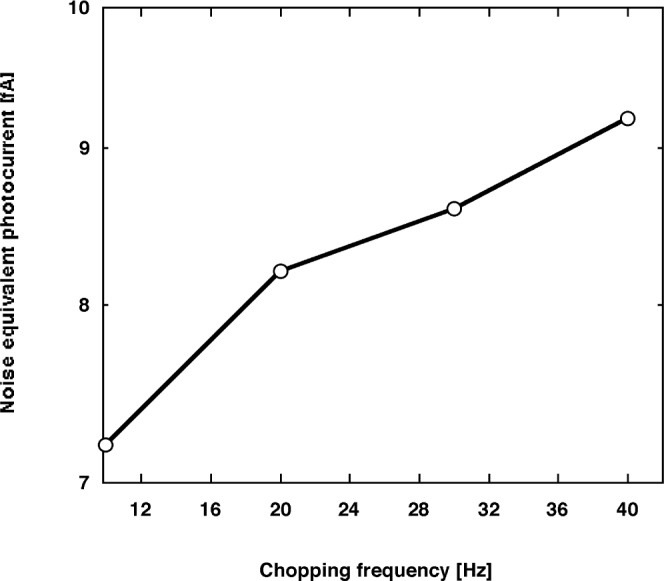
Measured noise floor vs chopping frequency of the trap radiometer at a signal gain of 10^8^ V/A.

The output total noise of the trap radiometer was measured in the dark between 10 Hz and 40 Hz when the signal gain was 10^8^ V/A and the signal roll-off was tuned to 400 Hz. The tuning was done by a 2 pF external capacitor connected parallel to the 10^8^ Ω feedback resistor. Figure 12 shows that the 7.2 fA noise-equivalent photocurrent at 10 Hz increased to 9.3 fA at 40 Hz. This result indicates that the effect of the increasing voltage gain with increasing frequency supersedes the decrease of the 1/*f* noise.

**Fig. 13:**
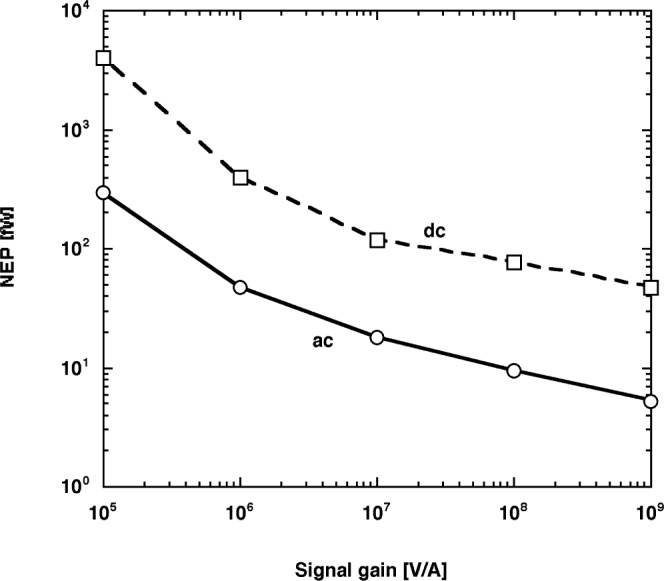
Comparison of the measured dc and ac noise-equivalent-power (NEP) values vs signal gain.

The total noise of the trap radiometer was measured at the optimum 10 Hz chopping frequency for all signal gain selections. An integrating time constant of 3.3 s was selected for the lock-in amplifier. The results, converted to noise equivalent power (NEP) using the 845 nm trap-detector responsivity, are shown with a solid curve in Fig. 13. The NEP is equal to the noise divided by responsivity.

### 3.4 Comparison of DC and AC Noise

The dc noise measured in the dark, also converted to NEP, is shown with the dashed curve in Fig. 13. The lowest NEP, equal to 5.2 fW, was measured in the ac mode at a signal gain of 10^9^ V/A. The curves show that the ac NEP is about an order of magnitude smaller than the dc NEP. According to these NEP results, the dynamic range of trap-radiometer No. 03 is between 5 × 10^−15^ W and 10^−2^ W. This range is larger than twelve decades. However, the output voltage produced by the signal should not be lower than 35 mV if a signal-to-noise ratio of 10^4^ is to be achieved. The 35 mV corresponds to 52 pW at 845 nm in the ac mode.

Table 2 shows the dc to ac input voltage-noise *V*_I_ ratios for the two highest signal gains of the trap radiometer. The ratios were calculated from the measured total noise *V*_T_ from Fig. 13 and the calculated voltage gains *A*_V_ (at dc and 10 Hz) from Fig. 11. The 1/*f* current noise and the Johnson noise were much smaller than the amplified 1/*f* input voltage noise, although the calculated input 1/*f* voltage noise *V*_I_ at 10^8^ Ω was 28 % higher (as compared to 10^9^ Ω) owing to some Johnson-noise contribution in the measured total noise. The 1/*f* input voltage noise decreased by about a factor of 50 when the signal frequency was changed from dc (less than 0.3 Hz) to 10 Hz. At the same time, as shown in Table 2, the increase in voltage gain from dc to 10 Hz was only a factor of 10. If we take the factor of 6 bandwidth difference between the dc and ac measurements into consideration, the one order of magnitude noise-floor difference in Fig. 13 can be understood. The results show that the highest sensitivity can be achieved at 10^9^ V/A signal gain selection in the ac measurement mode with a chopping frequency of 10 Hz. The relative standard uncertainty of these noise measurements was 18 %.

While the electrical bandwidth of the dc measurements was 0.3 Hz, that of the ac (10 Hz) measurements was six times smaller (0.05 Hz).

### 3.5 Calibration of the Current-to-Voltage Converter

For state-of-the-art radiant power, radiance, and irradiance responsivity calibrations [19, 20], the calibration accuracy of the photocurrent meter has to be similar to that of the spectral power responsivity and aperture area measurements. As discussed earlier, the values of the feedback resistances should determine the current-to-voltage conversion in a well designed converter. However, a feedback resistance is usually different from its nominal value. To achieve the required high accuracy requirement, primary level current-to-voltage gain calibrations are made against standard resistors. The standard resistors have temperature coefficients of less than 10^−5^ *R*/°C and are located in a temperature controlled and shielded box. They can be switched in decadic steps up to a maximum of 1 GΩ. The standard resistors can be serially connected to the input of the current-to-voltage converter. The source can be either a voltage or a current source. Both sources need high stability, but the voltage source does not need a large dynamic signal range. The feedback resistor of the converter can be calculated from voltage measurements at the input and output. The calibrated current-to-voltage converter can be used to calibrate other (test) current-to-voltage converters. A low drift and wide range current source, such as the Keithley Model 263 Calibrator/Current Source, can be switched to the inputs of either the standard or the test current-to-voltage converter while the converter output voltages are measured. This substitution-type calibration transfer can be used for all signal gain selections of the current-to-voltage converters. Using the above described two-step method, a 0.01 % level of uncertainty can be achieved in current-to-voltage gain calibrations.

## 4. Conclusion

The optical and electronic design of the new power and irradiance measuring tunnel-trap radiometer is described here in detail. This improved performance transfer standard is used to realize and/or hold the high accuracy spectral power, irradiance, and radiance responsivity scales of NIST [19, 20].

At present, trap detectors are not commercially available. Also, previously developed trap devices cannot satisfy the increasing requirements of modern spectral power and irradiance responsivity measurements. In order to transfer responsivity scales to applications with uncertainties similar to those of the primary standards, the national standard laboratories, including NIST, had to develop their own improved performance transfer standard trap devices [21, 22]. The performance of the tunnel-trap radiometer we developed earlier from six equal size S1337 photodiodes was improved. In the new optical and mechanical design, six photodiodes of two different sizes and an input aperture were tightly packed. A second light trap was attached to the radiometer output to absorb transmitted radiation, to minimize measurement of ambient light, and to simplify alignment and spectral responsivity calibrations. The stray radiation at the input was minimized as well. The separation between the aperture and the first photodiode was large enough to insert additional optical components, such as 10 mm long filter packages, without degrading the geometrical performance of the trap device.

Because of the improved optical-mechanical design and fabrication of the new trap radiometer, the 0.1 % deviation from the cosine function within 4° FOV (as performed on the previously made experimental device) decreased by a factor of five within a FOV of 5°. The 0.02 % deviation increased to about 0.05 % at 8° FOV.

Commercially available current meters cannot achieve the 0.01 % uncertainty requirement of trap-detector photocurrent measurements. It was necessary to design a current-to-voltage converter that is matched to the impedance of the trap detector package in order to perform the signal measurements with the required uncertainty.

The photodiodes in the trap-detector package were selected with similar shunt resistances to minimize voltage gain for the 1*/f* input voltage noise and drift of the operational amplifier. The voltage gain, the loop gain, and the signal gain were optimized for a signal (chopping) frequency of 10 Hz. A noise equivalent power of 47.4 fW was obtained in the dc measurement mode and 5.2 fW at 10 Hz signal frequency for a wavelength of 845 nm. The bandwidth was 0.3 Hz in the dc mode and 0.05 Hz in the ac mode. The measured one-order-of-magnitude noise-floor difference between dc and ac NEP was verified by a noise analysis. It was shown that the drift in the output voltage of the converter is roughly equal to the noise-produced output voltage (in the dc mode) if the temperature change of the operational amplifier is not larger than 1 °C.

As a result of optimizing the required electronic devices, a simple and inexpensive trap-radiometer could be implemented with a signal dynamic range from 5 × 10^−15^ W to 10^−2^ W. This dynamic range is larger than twelve decades.

Linearity, polarization-dependent responsivity, spatial-response uniformity, and the spectral power and irradiance responsivity of the triangular trap radiometer will be discussed in another publication.

## Figures and Tables

**Table 1:** Current-to-voltage converter feedback components and signal roll-off points for a silicon trap detector with 31 MΩ shunt resistance, 2 pF stray capacitance, resultant junction capacitance of 7.5 nF, and using operational amplifier OPA627BM

Feedback resistor (Ω)	Feedback capacitor (pF)	Signal roll-off frequency (Hz)	Compensation type
10^9^	0	80	None
10^8^	16	100	Partial
10^7^	160	100	Partial
10^6^	1600	100	Partial
10^5^	7480	213	Full
10^4^	7500	2123	Full

**Table 2:** DC input voltage-noise to ac input voltage-noise ratios calculated from the measured total noise and calculated voltage-gains

Signal Gain (V/A)	Total noise *V*_T_ (μV)		Voltage-gain *A*_V_		*V*_I_ = *V*_T_/*A*_V_ (μV)		*V*_I_(dc)/*V*_I_(ac)
	dc	ac	dc	ac	dc	ac	
10^9^	32.2	6.6	33.3	333	0.97	0.02	48.5
10^8^	5.24	0.9	4.23	42.3	1.24	0.021	59
